# Reduced plastid genomes of colorless facultative pathogens *Prototheca* (Chlorophyta) are retained for membrane transport genes

**DOI:** 10.1186/s12915-024-02089-4

**Published:** 2024-12-18

**Authors:** Kacper Maciszewski, Gabriela Wilga, Tomasz Jagielski, Zofia Bakuła, Jan Gawor, Robert Gromadka, Anna Karnkowska

**Affiliations:** 1 Institute of Evolutionary Biology, Faculty of Biology, Biological and Chemical Research Centre, University of Warsaw, Warsaw, Poland; 2 Institute of Parasitology, Biology Centre, Czech Academy of Sciences, České Budějovice, Czech Republic; 3 Department of Medical Microbiology, Institute of Microbiology, Faculty of Biology, University of Warsaw, Warsaw, Poland; 4 DNA Sequencing and Synthesis Facility, Institute of Biochemistry and Biophysics, Polish Academy of Sciences, Warsaw, Poland

**Keywords:** *Prototheca*, Plastid genomes, Colorless plastids, Chlorophyta

## Abstract

**Background:**

Plastids are usually involved in photosynthesis, but the secondary loss of this function is a widespread phenomenon in various lineages of algae and plants. In addition to the loss of genes associated with photosynthesis, the plastid genomes of colorless algae are frequently reduced further. To understand the pathways of reductive evolution associated with the loss of photosynthesis, it is necessary to study a number of closely related strains. *Prototheca*, a chlorophyte genus of facultative pathogens, provides an excellent opportunity to study this process with its well-sampled array of diverse colorless strains.

**Results:**

We have sequenced the plastid genomes of 13 *Prototheca* strains and reconstructed a comprehensive phylogeny that reveals evolutionary patterns within the genus and among its closest relatives. Our phylogenomic analysis revealed three independent losses of photosynthesis among the *Prototheca* strains and varied protein-coding gene content in their ptDNA. Despite this diversity, all *Prototheca* strains retain the same key plastid functions. These include processes related to gene expression, as well as crucial roles in fatty acid and cysteine biosynthesis, and membrane transport.

**Conclusions:**

The retention of vestigial genomes in colorless plastids is typically associated with the biosynthesis of secondary metabolites. In contrast, the remarkable conservation of plastid membrane transport system components in the nonphotosynthetic genera *Prototheca* and *Helicosporidium* provides an additional constraint against the loss of ptDNA in this lineage. Furthermore, these genes can potentially serve as targets for therapeutic intervention, indicating their importance beyond the evolutionary context.

**Supplementary Information:**

The online version contains supplementary material available at 10.1186/s12915-024-02089-4.

## Background

Plastids are eukaryotic organelles derived from cyanobacteria, whose most widely recognized and deeply studied function is photosynthesis [1, 2]. However, even though carrying the photosynthetic apparatus is their distinctive feature, a variety of biochemical pathways have been inherited by the extant plastids from their cyanobacterial ancestor, and their functions, such as heme, fatty acid, or amino acid biosynthesis, remain crucial constituents of the hosts’ metabolism [3–5]. As a result, the rather common loss of photosynthesis does not necessarily lead to the disappearance of the organelle—on the contrary, the so-called colorless plastids can be found in almost all known plastid-bearing lineages [4, 6], with only a handful of known cases of photosynthesis loss leading to a radically different outcome [7, 8].

All photosynthetic plastids and most non-photosynthetic ones carry their own genomes (plastomes, ptDNA), which are vestigial forms of the genome of the ancestral cyanobacteria. During the course of evolution, a vast majority of cyanobacterial genes have been lost or transferred to the nuclear genome of the host. This transfer, however, is not random, and certain genes, such as those encoding photosystem complex components, tend to be retained in the plastid genomes of all known plastid-bearing lineages [9]. With certain exceptions, even the most functionally reduced plastids retain genomes, whose coding contents are usually limited to up to several metabolically relevant protein-coding genes, acting as a constraint against complete genome loss, and a minimal transcription and translation apparatus [10–14]. Despite their small size and modest genetic repertoire, the genomes of colorless plastids can be important sources of insight into the evolutionary history and lifestyle of their host, as well as, in case of plastid-bearing parasites, even their vulnerabilities [3, 11, 15, 16].

To understand the plastid and plastid genome (plastome) evolution in secondarily non-photosynthetic organisms, comparative genomic and transcriptomic analyses with their photosynthetic relatives are the most common *modus operandi*. This applies both to lineages where photosynthesis losses have repeatedly occurred late in their evolution, such as orchids [17], dinoflagellates [8, 18] or the diatom genus *Nitzschia* [19], and those whose shift toward heterotrophy preceded their major radiation, such as apicomplexans [15, 20]. Close relatedness between organisms exhibiting vastly different lifestyles is often a hallmark of complex and captivating evolutionary paths, and a perfect example of that can be found among the green algal order Chlorellales. The relatives of the model green microalga *Chlorella* include a photosynthetic genus *Auxenochlorella*, as well as two secondarily non-photosynthetic genera – *Helicosporidium*, which are highly specialized gut parasites of insects [21–24], and *Prototheca*, which are predominantly free-living opportunistic pathogens of diverse vertebrates, including humans [25–28]. The evolutionary history of these organisms, especially their transitions between photosynthetic, parasitic, and free-living heterotrophic lifestyles, remains mysterious even with the availability of several genomic datasets [26, 29–31].

The aforementioned assemblage of *Auxenochlorella*,* Helicosporidium*, and *Prototheca*, collectively referred to as the AHP clade [32], constitutes a rather unique model for studying evolutionary transitions related to plastid reduction. In a recent work, it has been shown that photosynthesis was most likely lost several times independently in that clade [32], providing an excellent model group to study parallels in the reductive evolution accompanying the loss of photosynthesis in close relatives. This topic is particularly interesting because non-photosynthetic primary plastids often tend to adopt extreme forms, as demonstrated by cases of ptDNA inflation beyond the size observed in their photosynthetic relatives in certain green algae, such as *Leontynka pallida* or *Polytoma uvella* [33, 34], as well as complete ptDNA loss in other chlorophytes (*Polytomella parva*) [14] and even land plants (*Rafflesia* sp.) [35]. On the other hand, genomes of colorless plastids that are reduced in size and function, while retaining a rudimentary set of genes associated with metabolite synthesis and housekeeping functions, are more typical for the substantially better-studied secondary plastids, found e.g. in diatoms or apicomplexans [3, 36].

What is more, *Prototheca* and *Helicosporidium* are also among the extremely rare primary plastid-bearing pathogens, which makes the convergently similar form of their ptDNA to apicoplast genomes even more interesting. However, although *Prototheca* infections have been repeatedly observed in humans, dogs, and cows, its occurrence in a vast variety of other vertebrate hosts has been documented almost entirely in single case studies [27]; the transmission, infectivity, and mechanisms of pathogen-host interactions therefore remain unknown [37]. As demonstrated by the past studies of Apicomplexa, understanding the functions of vestigial plastids in parasites can not only provide key insights into their metabolic dependence on the host [38] but also uncover potential targets for therapeutical agents [39].

## Results and discussion

### Plastid-based phylogeny of the genus *Prototheca*

**Fig. 1:**
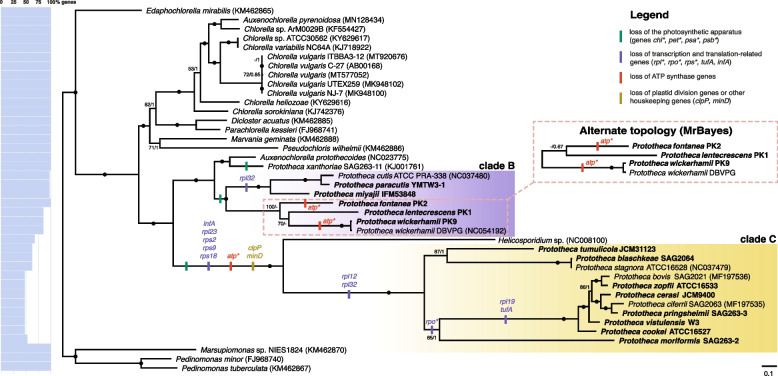
Phylogenomic analysis of the *Prototheca* and other Chlorellales with mapped gene losses on the respective branches. Tree shown is a maximum likelihood (ML) phylogeny of 40 marker genes from plastid genomes. The Bayesian inference phylogeny was congruent with ML with the exception of the relationships between *P. fontanea*, *P. lentecrescens*, and *P. wickerhamii*, and the incongruence is presented as an alternate topology. The phylogeny is based on a concatenated marker gene alignment of 24,003 unambiguously aligned sites under the model LG + F + I + G4. Black dots indicate maximal support for a particular node. When not maximal, only *a posteriori* > 0.5 and bootstrap support values > 50% are shown. Strain names in bold denote ptDNA sequences obtained in this study. NCBI GenBank accession numbers for sequences publicly available prior to this study are provided in brackets. The percentage of the total number of 40 plastid-encoded genes used in the construction of the data matrix, present in each of the taxa included, is shown on the horizontal bar plot on the left

**Fig. 2:**
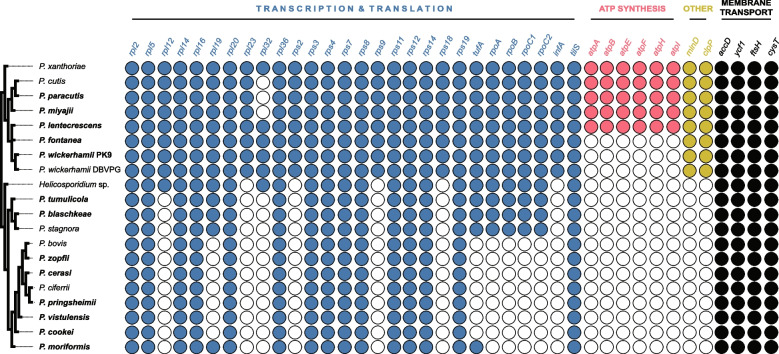
Distribution of various plastid genome functions across *Prototheca* spp. Note: the schematic phylogenetic tree on the left demonstrates solely the branching order of *Prototheca* clades; branch lengths are not to scale, and photosynthetic taxa have been removed for clarity

Plastid genome characteristics of *Prototheca* spp. are shown in Table 1. The plastid genome-based phylogenetic tree of *Prototheca* spp. and their relatives is shown in Fig. 1. Despite the overall high support for the reconstructed phylogeny, both estimated by Bayesian posterior probability and bootstrap support values, we observed one topological incongruency between the Bayesian and maximum likelihood reconstructions. In the ML reconstruction, *P. lentecrescens* branches off as a sister to *P. wickerhamii*, while *P. fontanea* branches off as sister to *P. lentecrescens* + *P. wickerhamii* clade*.* In the Bayesian tree, however, *P. lentecrescens* and *P. fontanea* form a clade of their own, branching off as sister to *P. wickerhamii*. The gene content of the plastid genomes of the *Prototheca* strains in question does not favor any of these two topologies, as they both imply two independent losses of the same gene set (the six-gene *atp* family) in closely related lineages. Therefore, with no scenario being more evolutionarily plausible than the other, we present the relationship of *P. wickerhamii*,* P. fontanea*, and *P. lentecrescens* as trichotomous in Fig. 2.

**Table 1:** Plastid genome characteristics of *Prototheca* spp

Organism	ptDNA size (bp)	Protein-coding genes	Accession no
*Prototheca xanthoriae* SAG 263–11	55,636	40	KJ001761
*Prototheca cutis* ATCC PRA-338	51,673	40	NC037480
*Prototheca wickerhamii* DBVPG	47,997	35	NC054192
*Prototheca stagnora* ATCC 16528	48,253	29	NC037479
*Prototheca bovis* SAG 2021	28,638	19	MF197536
*Prototheca ciferrii* SAG 2063	28,698	19	MF197535
***Prototheca paracutis* YMTW3-1**	51,694	40	PP291735
***Prototheca miyajii* IFM 53848**	53,237	40	PP291733
***Prototheca lentecrescens* PK1**	53,163	40	PP291732
***Prototheca fontanea* PK2**	46,196	34	PP291731
***Prototheca wickerhamii* PK9**	47,600	35	PP291739
***Prototheca tumulicola* JCM 31123**	49,137	26	PP291737
***Prototheca blaschkeae* SAG 2064**	46,294	30	PP291728
***Prototheca zopfii* ATCC 16533**	28,349	19	PP291740
***Prototheca cerasi* JCM 9400**	28,412	19	PP291729
***Prototheca pringsheimii* SAG 263–3**	28,370	19	PP291736
***Prototheca vistulensis* W3**	28,516	19	PP291738
***Prototheca cookei* ATCC 16527**	28,282	19	PP291730
***Prototheca moriformis* SAG 263–2**	38,525	22	PP291734

Taxa whose plastid genomes were first sequenced in this study are distinguished in bold

Regardless of the incongruency described above, both methods resolve the three main *Prototheca* clades identically, with the first clade (“*Prototheca* clade A”, represented solely by *P. xanthoriae* strain SAG 263–11*,* formerly classified as *P. wickerhamii* [25, 31]) branching off as sister to *Auxenochlorella protothecoides*, the second clade (encompassing *P. cutis*,* P. paracutis*,* P. miyajii*,* P. wickerhamii*,* P. fontanea*, and *P. lentecrescens*; further referred to as “*Prototheca* clade B”) branching as sister to the *A. protothecoides* + *P. xanthoriae* clade, and the third (encompassing all remaining species: *P. tumulicola*,* P. blaschkeae*,* P. stagnora*,* P. moriformis*,* P. bovis*,* P. zopfii*,* P. cerasi*,* P. ciferrii*,* P. pringsheimii*,* P. vistulensis*, and *P. cookei*; further referred to as “*Prototheca* clade C”) branching off as sister to *Helicosporidium* sp. As the last common ancestor of all *Prototheca* spp. is also the ancestor of *Helicosporidium* sp. and *Auxenochlorella protothecoides*, the genus *Prototheca* is therefore, by definition, polyphyletic, as suggested in the previous nuclear and mitochondrial gene-based phylogenies [27, 28].

Furthermore, it is clear from the plastid genome-based phylogeny presented above that all three *Prototheca* clades originate from ancestors which lost their photosynthetic capabilities independently. This result is also corroborated by an additional phylogeny of the AHP clade we reconstructed, based on 92 genes encoded in the nuclear genomes (Additional File 1: Fig. S1) suggesting at least three losses of photosynthetis in this clade. The nuclear gene-based tree was resolved with slightly different topology: the *Prototheca* clade B was split into two clades, and a subclade of *Prototheca* clade C, formed by *P. blaschkeae, P. stagnora* and *P. tumulicola* was split into three independent branches. Additionally, the branching pattern within clades was not congruent across all taxa. Nonetheless, this topology still supports the polyphyly of the genus *Prototheca* with respect to *Auxenochlorella* and *Helicosporidium,* with the sister relationships of *P. xanthoriae* and *A. protothecoides*, as well as *Helicosporidium* sp. and *Prototheca* clade C. Hence, the disparities between the observed branching patterns of nuclear and plastid phylogenies do not influence our interpretation of the order and numbers of plastid-encoded gene losses. Conflicting phylogenetic signals between plastid-encoded and nuclear-encoded genes have been reported in different plant groups [40–43]. The causes of these conflicts are often unclear, underlining the importance of analyzing both data sets. The differences in *Prototheca* tree topologies based on plastid and nuclear genes should therefore be further investigated to resolve the species tree within this group. However, to confirm these incongruences, and to better understand the differences in phylogenetic signals between plastid and nuclear genomes in *Prototheca*, a more comprehensive nuclear gene dataset based on complete nuclear genomes is required.

Still, due to vast discrepancies in sampling across the diversity of *Prototheca* spp. between the aforementioned phylogenies and the current work, we believe them to be impossible to compare in detail—for instance, the most recent *cytb*-based reconstruction included seventeen *P. ciferrii* sequences, while the species *P. paracutis* or *P. lentecrescens* have not been included at all, simply because they have not been described at the time [27, 44, 45].

### Independent losses of the photosynthetic apparatus shaped divergent paths of *Prototheca* plastid genomes

Interestingly, it is noticeable that the plastid genome contents differ substantially between the three *Prototheca* clades. While the ptDNA of *P. xanthoriae* (clade A) is both the largest (over 55.6 kbp) and *ex aequo* most gene-rich (40 protein-coding genes) of all *Prototheca* spp., the size and gene contents across the closely related *Prototheca* clade B are generally comparable, ranging from approximately 48.0 kbp and 35 protein-coding genes in *P. wickerhamii* to approximately 53.2 kbp and 40 protein-coding genes in *P. lentecrescens*. In contrast, the plastid genomes of the *Prototheca* clade C carry no more than 26 protein-coding genes, with many species (e.g., *P. zopfii*,* P. bovis*) having only 19. Moreover, the diminished gene content of the ptDNA in *Prototheca* clade C is not entirely proportional to their length – the plastid genomes of *P. tumulicola* and *P. stagnora* exceed 48.0 kbp in size, which would be within the range of *Prototheca* clade B, but their substantially smaller gene content indicates the elevated proportion of the non-coding regions in their plastid genomes. What is more, the ptDNA of *P. cookei* is below 28.3 kbp in size, which makes it, along with its closest relatives—*P. zopfii*,* P. ciferrii*,* P. bovis*,* P. pringsheimii*,* P. vistulensis*, and *P. cerasi*, all with ptDNA length below 29 kbp – the carrier of possibly the most reduced plastid genome among unicellular eukaryotes, with only a few species of mycoheterotrophic and parasitic plants reaching smaller ptDNA size and gene contents [12].

Furthermore, the reduction of coding contents of *Prototheca* plastomes is definitely not a symptom of their random decay in time, but a manifestation of divergence of function, as indicated by the retention or losses of complete gene operons or families, such as *atp* and *rpo*. Although the documentation for the loss of these two families in non-photosynthetic plastids is rather abundant, pointing toward the possibility of functional compensation for the missing *rpo* by nuclear RNA polymerases [46–49], the retention of ATP synthase subunits in *P. xanthoriae*,* P. cutis*,* P. paracutis*,* P. miyajii*, and *P. lentecrescens* indicates their capability for generating ATP or, alternatively, proton motor force across the plastid membrane. Although the ATP synthase subunits have been identified among the ptDNA contents of non-photosynthetic cryptophytes [50], diatoms [51], and even land plants [52], their role in absence of photosynthesis still awaits full explanation. Still, the presence of *atp* genes suggests that the plastids of the aforementioned five representatives of *Prototheca* carry out certain currently inscrutable metabolic processes absent from all the others. It might additionally be worth noting that five out of six plastid-encoded *atp* genes exhibit a significantly increased rate of evolution (expressed as *dN/dS* values; see Additional File 1: Table S2) in *Prototheca* compared to the photosynthetic Chlorellales, with the sole exception being the smallest subunit *atpH*, encoding only an approximately 80 amino acid long protein.

Bearing this functional diversity in mind, it is tempting to hypothesize whether the independent losses of photosynthesis in the ancestors of the three *Prototheca* clades might have been the cornerstone behind their divergence. A factor that certainly has to be taken into account is time—with the plastid genome of *P. xanthoriae* lacking only the photosynthetic apparatus (and therefore displaying rather “basic” reduction), compared to the phototrophic Chlorellales, and the ptDNA of many *Prototheca* clade C representatives being reduced to just a handful of genes (and therefore displaying “advanced” reduction), one could assume that the loss of photosynthesis in the clade C’s ancestor occurred earlier than in those of clade B and *P. xanthoriae.* Such a hypothesis, however, might be quite difficult to prove, as calibration of the evolutionary timeline would require insight into the fossil record. This, on the other hand, would be rather challenging not only due to the scarcity of adequately conserved fossilized remains of non-skeleton-forming unicellular eukaryotes, but also because of the near-identical morphology of all extant *Prototheca* species, which would make the phylogenetic placement of an extinct one nearly impossible.

### Membrane transport system components are the previously overlooked constraints against genome loss in *Prototheca* plastids

Despite the differences outlined above, plastid genomes of *Prototheca* spp. (and *Helicosporidium* sp.) also share a vast array of similarities—all examined species retained a common core set of 19 genes, covering the entire plastid gene repertoire of certain clade C representatives, such as the aforementioned *P. cookei*. This includes 15 genes involved in transcription and translation, but also a fatty acid synthesis-associated gene *accD*, and three membrane transport machinery components: *ycf1*, encoding the largest subunit of the protein translocation system TIC (TIC214), *cysT*, involved in sulfate ion import across the plastid membrane, and *ftsH* – a putative membrane translocation regulator (Fig. 2). Presence of the *ycf1* gene in the ptDNA of all studied *Prototheca* spp. is particularly interesting, as it has been documented to be quite frequently lost in various lineages of land plants (including photosynthetic ones), in addition to being a uniquely chlorophyte plastid-encoded gene, not found in rhodophytes, glaucophytes or any lineage bearing complex plastids [53]. Furthermore, identification of the *ycf1* gene may be challenging due to its fast-paced evolution [54], which is likely the reason why the biological role of its product in protein translocation across the inner envelope of plastids was only described a decade ago [53].

The available body of evidence suggests that the driving force behind genome retention in non-photosynthetic plastids is almost invariably the presence of indispensable plastid-encoded secondary metabolite synthesis pathway components [4, 11], with the only prominent exception to our knowledge being the dinoflagellate tRNA-fMet gene, encoded with the plastid genome, but directed to the mitochondria [55]. In contrast, the role of *ftsH* and *ycf1* is evidently the maintenance of transport mechanisms of a plastid compartment that serves almost entirely nucleus-encoded biosynthetic pathways, such as amino acid, heme, or fatty acid synthesis, demonstrated in past studies to be carried out in the vestigial plastids of *Helicosporidium* strain AT-2000 [21] and *Prototheca xanthoriae* strain SAG263-11 [25], and now found to have only two plastid-encoded components (*accD* and *cysT*) in total.

This work is not the first report of *ycf1* and *ftsH* retention in the genomes of non-photosynthetic plastids; both were reported in the previously studied plastid genomes of *Helicosporidium* and certain *Prototheca* spp., as well as the distantly related non-photosynthetic, although free-living chlorophyte *Polytoma uvella* [22, 32, 33, 56]. However, the potentially key role of *ycf1* may have been overlooked in the past, as the first reports of its retention in non-photosynthetic plastids [22] predate the discovery of this gene’s biological role [53, 57]—hence its name still suggests it to be a gene of unknown function. What is more, the bulk of studies on the roles of non-photosynthetic plastids is focused on secondary plastid-bearing lineages, such as apicomplexans, which do not possess a plastid-encoded *ycf1* [6].

The role of the plastid-encoded *ftsH* gene is substantially more complicated. The FtsH metalloprotease and its evolutionary relatives have been found to be involved a multitude of cellular functions, including chloroplast biogenesis [58] and turnover of thylakoid membrane-associated proteins [59] in photosynthetic plants, as well as cell division in prokaryotes, organelle division in *Cyanidioschyzon merolae* [60], and plastid-directed protein translocation in association with the TIC/TOC complex in both green and red primary plastids [61–63]. The relationship between the FtsH-like proteins and the TIC/TOC translocons is particularly interesting, as the hetero-hexameric complex of six different FtsH paralogs has been postulated to act as the ATPase motor facilitating the protein translocation [61, 64]. The FtsH-TIC/TOC interaction has been supported by evidence coming from the immunoprecipitation of TIC20 and FtsH in *Cyanidioschyzon merolae* [65], as well as *ftsH* knockout studies and a pulldown proteomic study of the TIC/TOC complex of *Chlamydomonas reinhardtii* [63, 66]. Furthermore, the sequences of *ftsH* and *ycf1* have also been demonstrated to be coevolving across a variety of green plastid-bearing taxa [61]. Thus, considering that *Prototheca* spp. plastids do not possess thylakoids [67] and do not carry the genes for crucial thylakoid-associated proteins, such as photosystem components, we are inclined to believe that it is the TIC/TOC-mediated protein translocation that remains the key role of FtsH in *Prototheca* and its relatives.

Nonetheless, the combination of non-transcription and translation-related genes retained in the plastid genomes of *Prototheca* (i.e., *accD*,* cysT*,* ftsH*,* minD*, and *ycf1*) is as interesting as it is unique among the nonphotosynthetic plastid-bearing organisms. As mentioned before, *ycf1* is not found in the genomes of any secondary plastids or red algal plastids. On the other hand, nonphotosynthetic green algae and land plants carrying plastid-encoded *ycf1*, i.e. the chlamydomonadaleans *Leontynka pallida* and *Polytoma uvella*, the liverwort *Aneura mirabilis*, and the orchid *Neottia nidus-avis*, are all missing *minD*, with the former two additionally missing *accD*, and the latter two missing *ftsH* [33, 34, 68, 69]. To the best of our knowledge, outside of the AHP clade, the aforementioned set of five genes is exclusively found in the plastid genomes of photosynthetic trebouxiophytes. Given that the genes missing from the ptDNA of various lineages are not always lost, but frequently undergo differential transfer to their respective hosts’ nuclei, this does not imply that other organisms cannot utilize the products of these genes in their plastid metabolism. To verify whether this is the case for the five genes mentioned above, a broad-scale transcriptomic survey of plastid-bearing eukaryotes is necessary, as the available data enabled us to identify the mitochondria-targeted copy of *ftsH* encoded in the nucleus in all *Prototheca* spp*.,* but not the nuclear counterparts of any of the remaining four plastid-encoded genes we investigated.

As a side note, we are not convinced that the retention of these particular genes in the plastid genomes of *Prototheca*, instead of them undergoing endosymbiotic gene transfer to the nucleus, is of any adaptational merit per se. Instead, we believe them to simply follow the same rules as all genes of endosymbiotic origin, i.e., that their genomic location is the resultant of a wide variety of evolutionary forces acting either toward retention in the organelle (e.g., to promote faster response in gene expression in response to redox shifts, as outlined in the CoRR hypothesis; see [9] and [70]) or transfer to the nucleus (e.g., to limit the energetic expense of maintaining a multi-copy organellar genome; see [71], or to restore the capability for recombination of genes originating from an asexually-replicating organelle; see [72]), with an immeasurable impact of random occurrences. Hence, by referring to certain genes as “constraints” against organellar genome loss, we do not imply them to be evolutionary constraints of global concern, but rather the constraints for a particular lineage in the present time frame, which is the product of its very unique evolutionary past.

### Unsolved mysteries: differential loss of the plastid-encoded ATP synthase and accelerated rate of evolution of miscellaneous genes

Moreover, the plastid genome contents that are not shared by all *Prototheca* spp. remain quite mysterious, especially the ATP synthase operon. Although its inconsistent presence in this genus has been pointed out before [32], the broader sampling of our study made it possible to observe that the entire *atp* gene set was differentially lost among *Prototheca* clade B, which has also been documented to occur in certain land plants, such as Orobanchaceae [47, 73], but in contrast with its consistent retention or loss in the descendants of all photosynthesis loss events in secondary plastids [32, 51]. In non-photosynthetic plastids, the proposed role of the ATP synthase complex is the hydrolysis of ATP to generate proton motive force across the inner plastid membrane, which is utilized for protein translocation by the twin-arginine translocase (Tat) system [51].

However, while the Tat system subunits have been identified in other non-photosynthetic lineages that retain ptDNA-encoded ATP synthase complex, e.g. *Leontynka pallida* (Chlorophyta) or *Cryptomonas paramecium* (Cryptophyta), the entire system seems to be absent both in plastid and nuclear genomes of all *Prototheca* investigated in this paper and previous works [32, 34, 50]. Therefore, the ATP synthase presence in some of the *Prototheca* spp. could be explained by the necessity to utilize ATP by the TIC/TOC translocon-associated FtsH motor mentioned earlier, or alternatively, by the existence of an unknown protein translocation system that relies on the proton gradient (under the assumption that the ATP synthase might be working in reverse, as mentioned earlier), or even a completely different, *Prototheca*-specific role of this complex in plastids, as proposed by Suzuki et al. [32]. Nonetheless, it is almost certain that there is a variability in plastid functions among *Prototheca* that cannot be fully explained by their plastome-encoded components.

Furthermore, the analysis of the rates of evolution of ptDNA-encoded genes between *Prototheca* clades yielded rather unexpected results (Additional File 1: Table S3). Among 25 analyzed genes, 7 (*ftsH*,* rpl16/19*,* rps8/14/19*, and *tufA*) displayed significantly increased *dN/dS* values in *Prototheca* clade C, compared to clade B; 17 others (*accD*,* rpl2/5/14/20/36*,* rpoA/B/C1/C2*,* rps3/4/7/11/12*,* tilS*, and *ycf1*) exhibited no difference between clades, but most surprisingly, one gene – *cysT* – has apparently undergone accelerated evolution in the *Prototheca* clade B, compared to clade C. This might be indicative of diversified evolutionary pressure toward different gene (and protein) sequence conservation between the *Prototheca* clades, with e.g. *ftsH* and *tufA* undergoing more constrained evolution in the *Prototheca* clade B, and *cysT* being more conserved in the clade C. It is also noteworthy that *ycf1* exhibited the overall highest rate of non-synonymous substitution of all plastid-encoded genes of *Prototheca* spp., corroborating the past observations on its fast-paced evolution [54].

Interestingly, despite the observed accelerated evolutionary rate and gene losses in the ptDNA of different *Prototheca* lineages, we have not identified a single symptom of pseudogenization, i.e., disruption of a reading frame in a discernible protein-coding gene. This stands in contrast with a wide array of past studies [73–75], in which pseudogenes have been frequently identified in non-photosynthetic primary plastid genomes, especially those of land plants, and have been considered the hallmark intermediate stages in the gradual reductive evolution of ptDNA.

## Conclusions

In this study, we obtained 13 new complete plastid genome sequences of *Prototheca* spp.—a paraphyletic assemblage of secondarily non-photosynthetic representatives of Chlorellales. We have demonstrated that despite having forfeited the photosynthetic apparatus three times independently and bearing highly variable coding contents, the plastid genomes of all *Prototheca* share the same key functions, which, apart from their own gene expression-related processes, include fatty acid and cysteine biosynthesis, as well as protein translocation across the organellar membrane. Additionally, our study is the first attempt to identify the patterns of differential reduction of ptDNA contents among the subclades of *Prototheca*, and to estimate the rate of evolution of the genes retained within the plastid genomes of different clades of *Prototheca* spp., which can be used as an indicator of the strength of purifying selection acting upon these genes. We observed certain evolutionary parallels in plastid genome evolution between our research subjects and various plastid-bearing lineages investigated in past works. However, the functional combination of the core ptDNA-encoded protein complement of *Prototheca* spp. including membrane translocation is rather unique.

Components of the pathways involved in secondary metabolite biosynthesis have been demonstrated in a variety of past studies to be the crucial factor behind the retention of vestigial genomes in colorless plastids of many eukaryotic lineages. The plastid membrane transport systems being an additional constraint against ptDNA loss in *Prototheca* and *Helicosporidium* makes these peculiar chlorophytes a prominent exception from the general paradigm, even considering that their retention in the organellar genome, as opposed to a possible transfer to the nucleus, is likely not adaptational. Still, the retention of genes unique to plastids in these opportunistic pathogens could be exploited in a clinical setting, as therapeutical agents targeting plastid transport machinery, such as the product of the gene *ycf1*, would likely pose minimal risk for the patients.

Nonetheless, we are convinced that the facultatively pathogenic inclinations of *Prototheca* have not been the main driving force between the repeated loss of photosynthesis. Instead, it seems more plausible that forfeiting photosynthesis accompanied the transition of these microorganisms to low-light habitats rich in total organic carbon, such as river sediments, demonstrated in a recent environmental survey [44] to be their important reservoir in nature. Still, as the *Prototheca* spp. seem to possess no obvious novel adaptations both to the benthic and the pathogenic lifestyle, it would be reasonable to perceive these organisms as ecological opportunists, losing excessive genetic and biochemical burdens over the course of evolution to limit the energetic expenses of survival.

Further genomic and transcriptomic studies are necessary to explain the diversity of ptDNA contents in *Prototheca* spp. and its possible correlation with diversified plastid metabolism. After all, despite its immense qualitative importance, the quantitative contribution of the plastid genome to the metabolism of the organelle is rather small, while the bulk of the organellar proteome, even if its evolutionary origin is endosymbiotic, comes from the genes presently encoded within the host nucleus. We believe that the key to unraveling the mystery behind the divergence among *Prototheca* may be understanding the role of the retained ATP synthase in these organisms’ plastids, and that the *Auxenochlorella/Helicosporidium/Prototheca* assemblage could become a promising model for future studies on divergent evolution of the endosymbiotic organelles, including, but not limited to the primary plastids.

## Methods

### Cultivation, DNA isolation, and sequencing

Nine strains of *Prototheca* spp. have been obtained from public culture collections indicated by their names where applicable: SAG—Culture Collection of Algae (Sammlung von Algenkulturen), University of Göttingen, Germany; JCM (+ strain YMTW3-1)—Japan Collection of Microorganisms, RIKEN BioResource Research Center, Tsukuba, Japan; ATCC—American Type Culture Collection, Manassas, VA, USA; IFM—Research Center for Pathogenic Fungi and Microbial Toxicoses (formerly Institute of Food Microbiology), Chiba University, Japan. Additionally, four *Prototheca* strains (PK1, PK2, PK9, and W3) were obtained from a private collection of the Department of Medical Microbiology (Institute of Microbiology, Faculty of Biology, University of Warsaw, Poland). All strains were cultured on Sabouraud Dextrose Agar (SDA) plates (Becton Dickinson, USA) and their DNA isolation was performed according to the optimized protocol based on homogenization with glass beads, outlined in [76]. The cell pellet from culture medium was suspended in 750 µL of extraction buffer (2% Triton-X100, 1% SDS, 100 mM NaCl, 10 mM Tris–HCl pH 8.0, 1 mM EDTA) and cell lysis was achieved by pulverization with 0.4–0.6 mm diameter glass beads (Sartorius AG, Göttingen, Germany), in a 1:1 ratio, in a tissue lyser (TissueLyser II; Qiagen, Hilden, Germany) at 20 Hz for 15 min. The disrupted sample was then transferred into a 5-mL microcentrifuge tube. The glass beads were washed 4 times with 500 µL of extraction buffer, and the washes were pooled. Cell lysis was continued with the addition of proteinase K (160 µg/mL) and incubation at 56 °C for 1 h. In the next step, 10% CTAB/0.5 M NaCl solution was added, followed by 10 min of incubation at 65 °C. The lysate was further extracted with an equal volume of Phe/Chl/IAA (25:24:1) and DNA was precipitated with 0.7 volume of isopropanol, followed by centrifugation (20 min, 14,000 rpm, RT), and washing with 1 mL of 70% ethanol. The DNA was air-dried and resuspended in 200 µL of TE buffer with RNAse A (50 µg/ml) and incubated at 37 °C for 30 min. DNA was finally centrifuged (5 min, 14,000 rpm, RT), and the clear supernatant was collected in a new tube. All DNA samples were sequenced using the Illumina MiSeq PE300 platform, with a 600-cycle chemistry kit.

### Quality control and genome assembly

Quality control of the obtained reads was carried out using FastQC v0.11.5 [77]. Adapter removal and trimming were performed using Trimmomatic v0.32 [78] using default parameters. The initial assembly was carried out using SPAdes v3.11.1 [79], and the outputs were analyzed to assess their general quality using Quast v5.0.2 [80]. The detection of potential contamination was done using Blobtools v1.1 [81].

Among the assembled contigs, plastid genome-derived sequences were identified using Tiara v1.01 [82], supplemented by BLASTn searches [83] using publicly available ptDNA sequences of *Prototheca* spp. as queries. The largest identified plastid genome fragments in each assembly were extracted and subsequently used as seeds for the final ptDNA assembly using NOVOPlasty v4.3 [84]. Circularized plastid genomes were recovered in all 13 datasets.

### Plastid genome annotation and visualization

Automatic annotation of *Prototheca* plastid genomes was carried out using Geneious Prime v2022.1.1 software (https://www.geneious.com) using Live Annotate & Predict toolkit (Find ORFs and Annotate From… features), utilizing a manually constructed database of published plastid genomes of *Prototheca* spp., *Chlorella* spp., *Auxenochlorella* spp., and *Parachlorella kessleri*. Identities of all protein-coding gene sequences were confirmed by alignment with the NCBI non-redundant protein database (NCBI-nr) via BLASTX algorithm [83], with the PFAM 35.0 protein families’ database (pfam.xfam.org) using the browser-accessible internal HMM search feature [85], and using the HHpred browser-accessible toolkit (toolkit.tuebingen.mpg.de; [86]). Additionally, genome assemblies were surveyed using a bi-directional BLAST search for nuclear copies of certain plastid-encoded genes. Plastid genome maps were generated using the OGDraw v1.3.1 online tool [87].

### Plastid genome-based phylogenomic analysis

Orthologs of 79 protein-coding genes were extracted from the 40 annotated ptDNA sequences of *Prototheca* and their closest relatives, including the 13 *Prototheca* strains analyzed in this work, six published plastid genomes of *Prototheca* (see Table 1), 11 published plastid genomes of *Chlorella,* two published plastid genomes of *Auxenochlorella*, two published plastid genomes of *Pedinomonas*, and the published plastid genomes of singular representatives of *Helicosporidium* sp., *Marsupiomonas* sp., *Dicloster acuatus*,* Marvania geminata*,* Parachlorella kessleri*, and *Pseudochloris wilhelmii* (see Additional File 1: Table S1)*.* All coding sequences were translated into amino acid sequences, aligned using the L-INS-I method in MAFFT v7.310 [88], trimmed via trimAl v1.4 [89], and concatenated using catsequences script (https://github.com/ChrisCreevey/catsequences) to produce data matrix with a total length of 33,713 amino acids. Genes that were not found in any of the analyzed *Prototheca* species (i.e., encoding photosynthesis-related proteins) were removed, resulting in a final matrix containing 40 genes with a total length of 24,003 amino acids. The percentage of genes missing from the final matrix for each taxon is depicted in Fig. 1.

The concatenated alignment was used as the input for phylogenetic analyses via the maximum likelihood method implemented in IQ-TREE v2.0.6 software [90], and via the Bayesian inference method implemented in MrBayes v3.2.6 [91]. Maximum likelihood phylogeny reconstruction used a partitioned matrix with LG + F + I + G4 substitution model, which was determined empirically as best-fitting via *-m TEST* followed by *-mset LG* + *G4,LG* + *C10,LG* + *C20,LG* + *C30,LG* + *C40,LG* + *C50,LG* + *C60* parameter, and 1000 non-parametric bootstrap replicates. The Bayesian reconstruction used a non-partitioned dataset with a preset sequence evolution model (*invgamma*), with 1,000,000 generations (incl. 250,000 generations burn-in), after which convergence of the four Markov chains was achieved at average standard deviation of split frequencies of 0.002049. Both methods yielded mostly congruent tree topology, with local divergence in topologies described in further detail in the “Results and discussion” section.

### Nuclear phylogenomic analysis

Orthologs of 255 nuclear genome-encoded genes, constituting the eukaryota_odb10 database, were identified in the genomic assemblies of *Prototheca, Chlorella*, *Auxenochlorella*, and *Helicosporidium* spp. using BUSCO v5.7.1 [92]. Single-copy genes were extracted and aligned using the L-INS-I method in MAFFT v7.310 [88]. 92 alignments containing sequences from at least 75% of the analyzed *Prototheca* spp. were concatenated using the catsequences script (https://github.com/ChrisCreevey/catsequences) to produce a raw data matrix with a total length of 55,187 amino acids. The raw dataset was subsequently trimmed via trimAl v1.4 [89] at gap threshold (-gt) 0.8 to produce the final data matrix with a total length of 19,739 amino acids. The concatenated alignment was used as the input for phylogenetic analyses via maximum likelihood method implemented in IQ-TREE v2.0.6 software [90] with LG + F + I + G4 substitution model, which was determined empirically as best-fitting via *-mset LG* + *G4,LG* + *C10,LG* + *C20,LG* + *C30,LG* + *C40,LG* + *C50,LG* + *C60* parameter, and 1000 non-parametric bootstrap replicates.

### Estimation of evolutionary rate

Codon alignments of 25 plastid protein-coding genes were prepared using PAL2NAL v14 software [93]. Rates of synonymous and non-synonymous substitutions (*dN/dS*) for all gene alignments were calculated using the CodeML tool implemented in the PamlX v1.3.1 toolkit [94]. Mean *dN/dS* values were calculated for two groups: *Prototheca* clade B (7 taxa) and clade C (11 taxa) for all 25 protein-coding genes identified in the ptDNA sequences obtained for these taxa, and compared using two-sided Mann–Whitney *U*-test implemented in Social Science Statistics calculator (online tool; https://www.socscistatistics.com/tests/mannwhitney/). *Prototheca* clade A was not included in this analysis, as it comprises only one taxon.

## Supplementary Information

Additional file 1: Fig. S1. Maximum likelihood phylogenomic analysis of the * Prototheca* and other Chlorellales based on 92 eukaryotic nuclear gene markers; Table S1. Table S1. NCBI accession numbers of published non- *Prototheca* plastid genome assemblies used in this study; Table S2. Comparison of the rate of evolution (*dN/dS*) of ATP synthase subunits in the plastid genomes of * Prototheca* spp. and photosynthetic Chlorellales; and Table S3. Comparison of the rate of evolution (*dN/dS*) of protein-coding genes in the plastid genomes of *Prototheca* clades B & C.

## Data Availability

Plastid genome sequences obtained in this study have been deposited in the NCBI GenBank database under the accession numbers: PP291728-PP291740. Additional datasets generated and/or analyzed during the current study, including the plastid genome-derived concatenated protein alignments, are available in the FigShare repository under the following link: 10.6084/m9.figshare.24973665 [95].

## Abbreviations

AHP: *Auxenochlorella/Helicosporidium/Prototheca*
ATP: Adenosine triphosphate
CoRR: Colocation for redox regulation
kbp: Thousand base pairs
ML: Maximum likelihood
ptDNA: Plastid genome
Tat: Twin-arginine translocation
TIC/TOC: Translocon on the inner/outer chloroplast membrane
