# Supplementary material for: Reduced plastid genomes of colorless facultative pathogens Prototheca (Chlorophyta) are retained for membrane transport genes
Source: BMC Biol. 2024 Dec 18;22:294. doi: 10.1186/s12915-024-02089-4 (PMC11657870; doi:10.1186/s12915-024-02089-4)
Supplement: Supplementary file 1 — Additional file 1: Fig. S1. Maximum likelihood phylogenomic analysis of the Prototheca and other Chlorellales based on 92 eukaryotic nuclear gene markers; Table S1. Table S1. NCBI accession numbers of published non- Prototheca plastid genome assemblies used in this study; Table S2. Comparison of the rate of evolution (dN/dS) of ATP synthase subunits in the plastid genomes of Prototheca spp. and photosynthetic Chlorellales; and Table S3. Comparison of the rate of evolution (dN/dS) of protein-coding genes in the plastid genomes of Prototheca clades B & C. [file 12915_2024_2089_MOESM1_ESM.docx]

**Reduced plastid genomes of colorless facultative pathogens *Prototheca* (Chlorophyta) are retained for membrane transport genes**

Kacper Maciszewski^1,2^, Gabriela Wilga^1^, Tomasz Jagielski^3^, Zofia Bakuła^3^, Jan Gawor^4^, Robert Gromadka^4^, Anna Karnkowska^1^*

**^1^**Institute of Evolutionary Biology, Faculty of Biology, Biological and Chemical Research Centre, University of Warsaw, Poland;

^2^Institute of Parasitology, Biology Centre, Czech Academy of Sciences, České Budějovice, Czech Republic

^3^Department of Medical Microbiology, Institute of Microbiology, Faculty of Biology, University of Warsaw, Poland

^4^DNA Sequencing and Synthesis Facility, Institute of Biochemistry and Biophysics, Polish Academy of Sciences, Warsaw, Poland

*corresponding author; e-mail: a.karnkowska@uw.edu.pl

**Supplementary Figures**


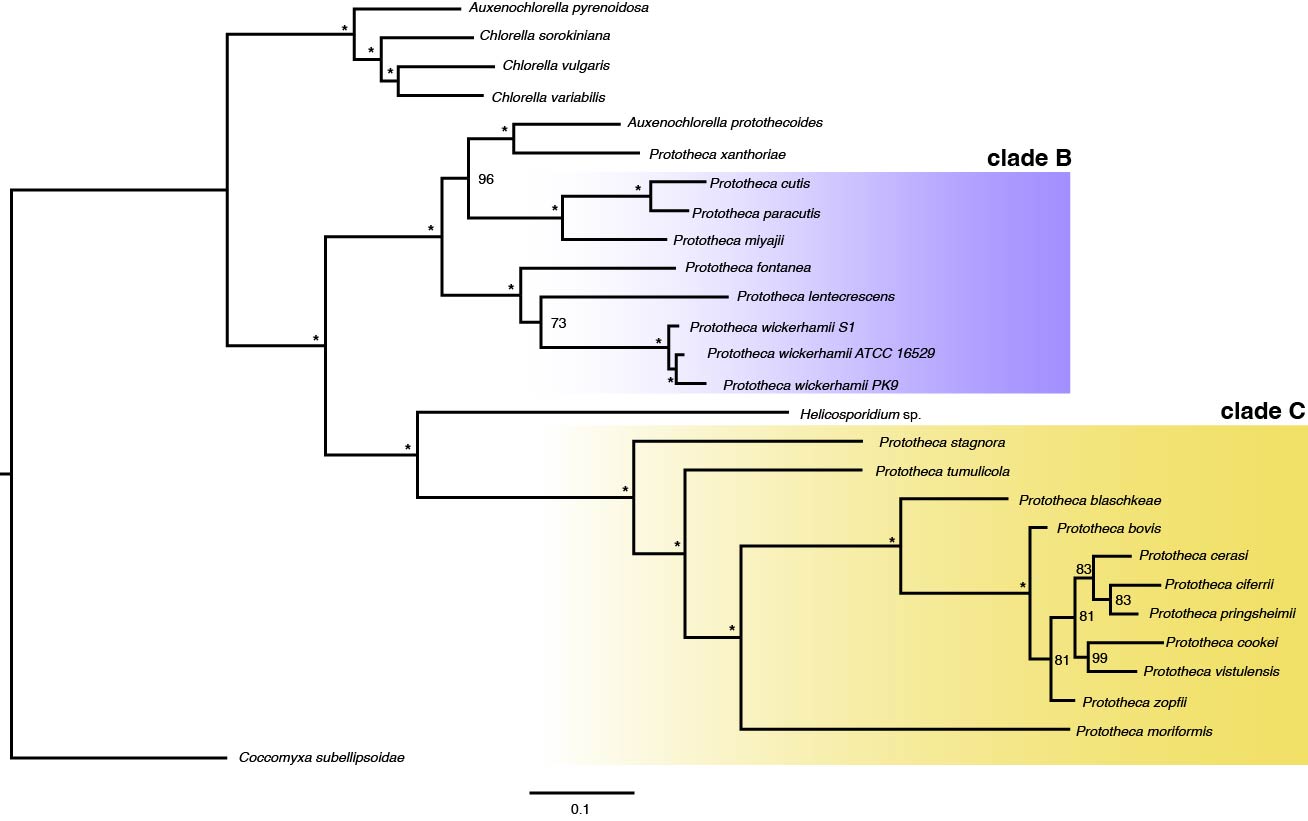


**Fig. S1.** Phylogenomic analysis of the Prototheca and other Chlorellales. Tree shown is a maximum likelihood (ML) phylogeny based on 92 eukaryotic nuclear gene markers (BUSCOs; 19,739 positions). Stars indicate maximum bootstrap support for a particular node. When not maximal, only bootstrap support values >50% are shown.

**Supplementary Tables**

**Table S1.** NCBI accession numbers of published non-*Prototheca* plastid genome assemblies used in this study.

| **Organism** | **Accession no.** |
| --- | --- |
| *Auxenochlorella protothecoides* | NC023775 |
| *Auxenochlorella pyrenoidosa* FACHB-5 | MN128434 |
| *Chlorella heliozoae* | KY629616 |
| *Chlorella sorokiniana* isolate 1230 | KJ742376 |
| *Chlorella* sp. ArM0029B | KF554427 |
| *Chlorella* sp. ATCC 30562 | KY629617 |
| *Chlorella variabilis* isolate NC64A | KJ718922 |
| *Chlorella vulgaris* | NC001865 |
| *Chlorella vulgaris* C27 | AB001684 |
| *Chlorella vulgaris* clone ITBBA3-12 | MT920676 |
| *Chlorella vulgaris* NJ-7 | MK948100 |
| *Chlorella vulgaris* UTEX 259 | MK948102 |
| *Dicloster acuatus* SAG 41.98 | KM462885 |
| *Edaphochlorella mirabilis* SAG 38.88 | KM462865 |
| *Helicosporidium* sp. ex-*Simulium jonesi* | NC008100 |
| *Marsupiomonas* sp. NIES 1824 | KM462870 |
| *Marvania geminata* SAG 12.88 | KM462888 |
| *Parachlorella kessleri* SAG 211-11g | FJ968741 |
| *Pedinomonas minor* UTEX LB 1350 | FJ968740 |
| *Pedinomonas tuberculata* SAG 42.84 | KM462867 |
| *Pseudochloris wilhelmii* SAG 1.80 | KM462886 |

**Table S2.** Comparison of the rate of evolution (dN/dS) of ATP synthase subunits in the plastid genomes of Prototheca spp. and photosynthetic Chlorellales.

| **Gene** | **Mean dN/dS ± st. dev.**  (top: photosynthetic Chlorellales; bottom: *Prototheca* spp.) | **Rate of evolution comparison** | Mann-Whitney *p*-value (significance) |
| --- | --- | --- | --- |
| *atpA* | 0.0677 ± 0.0331  0.243 ± 0.0662 | increased in *Prototheca* | 8.87·10^-8^ (***) |
| *atpB* | 0.0573 ± 0.0233  0.194 ± 0.0507 | increased in *Prototheca* | 7.56·10^-8^ (***) |
| *atpE* | 0.154 ± 0.0785  0.819 ± 0.222 | increased in *Prototheca* | 7.56·10^-8^ (***) |
| *atpF* | 0.174 ± 0.118  0.978 ± 0.306 | increased in *Prototheca* | 1.24·10^-7^ (***) |
| *atpH* | 0.117 ± 0.0490  0.135 ± 0.0515 | no difference | 0.189 |
| *atpI* | 0.0973 ± 0.0438  0.268 ± 0.0627 | increased in *Prototheca* | 1.65·10^-7^ (***) |
|  |  |  |  |

**Table S3.** Comparison of the rate of evolution (dN/dS) of protein-coding genes in the plastid genomes of Prototheca clades B & C.

| **Gene** | **Mean dN/dS ± st. dev.**  (top: clade B; bottom: clade C) | **Rate of evolution comparison** | Mann-Whitney *p*-value (significance) |
| --- | --- | --- | --- |
| *accD* | 0.277 ± 0.109  0.423 ± 0.260 | no difference | 0.0988 |
| *cysT* | 0.428 ± 0.144  0.167 ± 0.132 | clade B > clade C | 7.30·10^-6^ (***) |
| *ftsH* | 0.395 ± 0.0985  0.755 ± 0.360 | clade C > clade B | 1.12·10^-3^ (**) |
| *rpl2* | 0.216 ± 0.0552  0.181 ± 0.0797 | no difference | 0.0505 |
| *rpl5* | 0.322 ± 0.0696  0.394 ± 0.297 | no difference | 0.446 |
| *rpl14* | 0.191 ± 0.0426  0.159 ± 0.0899 | no difference | 0.131 |
| *rpl16* | 0.107 ± 0.0234  0.155 ± 0.0513 | clade C > clade B | 8.08·10^-5^ (***) |
| *rpl19* | 0.389 ± 0.0788  0.709 ± 0.0911 | clade C > clade B | 3.18·10^-4^ (***) |
| *rpl20* | 0.419 ± 0.0897  0.387 ± 0.236 | no difference | 0.190 |
| *rpl36* | 0.457 ± 0.489  0.638 ± 0.608 | no difference | 0.293 |
| *rpoA* | 0.529 ± 0.236  0.544 ± 0.472 | no difference | 0.271 |
| *rpoB* | 0.404 ± 0.400  0.296 ± 0.257 | no difference | 0.163 |
| *rpoC1* | 0.389 ± 0.0711  0.478 ± 0.414 | no difference | 0.0158 |
| *rpoC2* | 0.389 ± 0.0851  0.311 ± 0.269 | no difference | 0.0774 |
| *rps3* | 0.483 ± 0.0981  0.554 ± 0.342 | no difference | 0.0755 |
| *rps4* | 0.326 ± 0.121  0.523 ± 0.300 | no difference | 0.0110 |
| *rps7* | 0.476 ± 0.129  0.624 ± 0.377 | no difference | 0.134 |
| *rps8* | 0.433 ± 0.147  0.852 ± 0.480 | clade C > clade B | 2.50·10^-3^ (**) |
| *rps11* | 0.531 ± 0.173  0.571 ± 0.363 | no difference | 0.129 |
| *rps12* | 0.142 ± 0.0271  0.232 ± 0.121 | no difference | 0.0235 |
| *rps14* | 0.177 ± 0.0405  0.521 ± 0.200 | clade C > clade B | 6.54·10^-6^ (***) |
| *rps19* | 0.152 ± 0.0464  0.362 ± 0.196 | clade C > clade B | 8.06·10^-5^ (***) |
| *tilS* | 0.625 ± 0.194  0.599 ± 0.261 | no difference | 0.404 |
| *tufA* | 0.126 ± 0.0346  0.184 ± 0.0291 | clade C > clade B | 5.01·10^-3^ (*) |
| *ycf1* | 0.721 ± 0.185  0.858 ± 0.414 | no difference | 0.048 |
|  |  |  |  |
